# Kimberly A. Plomp, Charlotte A. Roberts, Sarah Elton and Gillian R. Bentley, eds., *Palaeopathology and Evolutionary Medicine: An Integrated Approach*

**DOI:** 10.1093/emph/eoac032

**Published:** 2022-08-19

**Authors:** Clark Spencer Larsen

**Affiliations:** Department of Anthropology The Ohio State University Columbus, OH, USA

Editors Kimberly A. Plomp, Charlotte A. Roberts, Sarah Elton and Gillian R. Bentley have developed an impressive volume focusing on the integration of paleopathology—the study of disease, health and the challenges to health in the past—and evolutionary medicine—the study of health in an evolutionary context. These two fields share an interest in past and present health. Paleopathology has its origins in the identification and diagnosis of skeletal and dental pathology as documented in human remains in all manner of preservation, ranging from mummified bodies to skeletons. Evolutionary medicine applies the science of evolutionary biology to interpret, prevent and treat disease. The book makes clear that the integration of paleopathology and evolutionary medicine offers new opportunities for the development of a more informed understanding of health and well-being, including, but not limited to, aging, reproductive health, immune function, inflammation, microbiomes, and diet and nutrition. Via the presentation of 18 chapters written by disciplinary experts, the book successfully integrates the two fields, giving both new strengths and revised aspirations in addressing common goals.

As noted by the editors in the lead chapter, ‘What’s It All About’, a primary goal of medical practitioners is to both understand and improve health now and in the future. Paleopathology and evolutionary medicine are built on the premise that current health conditions have deep temporal contexts. The record of past health, behavior and living circumstances is within the frameworks of bioarchaeology and paleopathology. Bioarchaeology is the study of human remains in archaeological contexts. Paleopathology is the study of ancient diseases viewed broadly. Both bioarchaeology and paleopathology are contextualized via reconstruction of health and the record of life experiences in the past. This expansive and complex record pertains to the roles of population size, density and relative permanence, degree of mobility, exposure to pathogens, diet and nutrition, and much more that collectively shape health and wellbeing. Bioarchaeology seeks to understand the circumstances of the lived experience and its impact on the skeleton and dentition—diet and nutrition, population size and density, mobility, workload and activity patterns, and range of other factors that created the health and behavioral environments of human ancestors and all of us living today. A great strength of this book is its success in recognition of shared interests that cross-disciplinary boundaries to pursue questions about health in past. Moreover, the contributions make clear that the broader understanding of health and health outcomes in the present requires documentation of the record of deep time, records that can only be accessed via paleopathology and bioarchaeology.

The allied fields of paleopathology (e.g. see Ref. [1]) and bioarchaeology (e.g. see Ref. [2]) present the broad perspective and context for the development of a deeper understanding of many of today’s health and behavioral challenges. Simply, the ‘narrow lens’ of the present is insufficient in scale and duration for tackling current and future health challenges. These allied fields give important insights into life as we know it today, via the remarkably large and growing picture of infectious disease and inadequate nutrition for much of the world’s eight billion occupants. Our knowledge of undernutrition and unprecedented levels of exposure to long-existing infectious diseases (e.g. leprosy, tuberculosis, treponematosis) is available owing to the extraordinary developments in paleopathology. I am especially impressed with the depth of coverage of the book as it pertains to a wide range of health conditions and the attention that it has been given to the new and growing understanding of disease offered by paleopathology and bioarchaeology. Both present important opportunities for investigating the evolution of the human condition, and the development of a broader understanding of the circumstances in the past that have led to the remarkable challenges the world is facing in the 21st century, including the rise of novel infectious diseases, unprecedented population growth and circumstances involving access to medical care and adequate nutrition.

The record of physiological disruption resulting from impoverished environmental conditions is central to the study of health and wellbeing. Although non-specific in definition, physiological disruption applies to a wide range of situations, including but not limited to insufficient nutrition, exposure to pathogens and other factors that challenge health. Temple and Edes, for example, present both a historical context for the study of stress and its application to bioarchaeology. Their discussion offers the kind of unifying approach that joins past and present and suggests clear implications for health challenges in the future. Past contextualizes present in fundamental ways.

The book covers considerable territory, including topics of famine and how nutritional deficiencies play out in the evolution of human developmental processes, spinal health, birth and mortality, dietary health, oral health and its connection with the oral microbiome and systemic health, plague, leprosy, tuberculosis, parasitic infection, cardiovascular disease, cancer, physiological stress, metabolic diseases, traumatic injuries, shared human and animal pathogens, and outcomes that provide a new and more informed meaning to health and well-being on a comprehensive scale. A strength of the book is its context presented in Chapter 1, including especially the three questions posed by editors Plomp, Roberts, Elton and Bentley, namely (i) How does paleopathology research on specific topics contribute to the understanding of the evolution of health and disease?; (ii) How does paleopathology offer a unique perspective and new understanding of these topics?; and (iii) How does this new knowledge contribute to modern clinical research and medicine?

In all ways, the book is effective in addressing these questions. I would have liked to have seen a more comprehensive discussion and more examples of bioarchaeological contextual approaches in the discussion of disease and health. Skeletons mean little without the broader context-based perspective provided by the archaeological record that represents the dietary, behavioral and settings generally where skeletal remains have been recovered and subsequently investigated. Bioarchaeology and paleopathology are both the human remains and their contexts. The contributors to the book are testing a single general hypothesis, namely that the collaboration and engagement of the emerging or otherwise maturing fields of evolutionary medicine will help us better understand the relative success of humans in the past, laying the foundation for our lives and challenges in the 21st century. The book offers numerous ways of testing the hypothesis and providing direction for building a foundation for a bright, new chapter in the natural and health sciences involving an evolutionary context. As so well stated in Jane Buikstra’s Afterward, ‘…it is important to think globally about health, interrogating the past in a framework that employs evolutionary principles…(and) drawing together disciplines that should comprise the integrated anchor for this initiative’.
